# Unveiling the influences of P fertilization on bioactive compounds and antioxidant activity in grains of four sorghum cultivars

**DOI:** 10.1371/journal.pone.0311756

**Published:** 2024-10-31

**Authors:** Mohammed Elsafy, Nouralhuda A. J. Tia, Khitma A. Sir Elkhatim, Mazahir H. Othman, Amro B. Hassan, Mahbubjon Rahmatov, Tilal Sayed Abdelhalim

**Affiliations:** 1 Department of Plant Breeding, Swedish University of Agricultural Sciences (SLU), Alnarp, Sweden; 2 Department of Plant Nutrition, Soil and Land Use Center, Agricultural Research Corporation, ARC, Wad Medani, Sudan; 3 Biotechnology and Biosafety Research Center, Agricultural Research Corporation, ARC, Shmbat, Sudan; 4 Department of Food Science and Nutrition, College of Food and Agricultural Sciences, King Saud University, Riyadh, Saudi Arabia; 5 Environment and Natural Resource and Desertification Research Institute (ENDRI), National Center for Research, Khartoum, Sudan; KGUT: Graduate University of Advanced Technology, ISLAMIC REPUBLIC OF IRAN

## Abstract

**Backgrounds:**

Phosphorus is a critical nutrient in agriculture, influencing plant growth and nutritional quality.

**Objectives:**

This study, uniquely designed to investigate the effects of phosphorus (P) fertilization levels, sorghum cultivars, and growing locations on phytochemical content and antioxidant activity in sorghum grains, employed four sorghum cultivars (Hakeka, P954063, Tabat, and Tetron) grown under three P levels (0P, 1P, 2P) in two locations (Gezira and White Nile) in Sudan.

**Methods:**

In this study, four sorghum cultivars were grown in two distinct locations in Sudan, employing a split-plot design with three (P) fertilization levels. P was applied as triple super phosphate directly with the seeds, and additional fertilization included urea applied in two split doses. At physiological maturity, representative sorghum panicles were harvested, processed, and analyzed for bioactive compounds and antioxidant activities using standard extraction and quantification techniques such as Folin-Ciocalteu for phenolics and colorimetric flavonoid assays. Antioxidant activities were assessed through various assays, including DPPH and FRAP. Statistical analyses were performed using a three-way ANOVA to examine the effects of cultivar, P level, and location on the measured parameters, supplemented by multivariate analysis to further elucidate the interactions between these factors.

**Results:**

Significant interactions (p<0.001) were observed among cultivars, P levels, and locations for total phenolic content (TPC), total flavonoid content (TFC), carotenoids, tannins, and various antioxidant activity measures (DPPH, FRAP, ABTS, TRP, H2O2). P fertilization significantly increased all measured phytochemicals and antioxidant activities compared to non-treated cultivars, except for H2O2, which decreased with P application. Among cultivars, Hakeka consistently exhibited the highest TFC, carotenoid content, and antioxidant activities (DPPH, FRAP, TRP, ABTS), particularly at the 2P level. P954063 showed the highest TPC and tannin concentrations. Tetron generally had the lowest phytochemical and antioxidant levels. White Nile showed higher TPC, carotenoids, DPPH, FRAP, TRP, and ABTS levels, while Gezira had higher TFC, tannins, and H2O2 concentrations. The impact of phosphorus fertilization often varies between locations. Strong positive correlations were found between TPC and all antioxidant assays (r = 0.68–0.90) and total carotenoids and antioxidant activities (r = 0.73–0.93).

**Conclusions:**

This study recommended cultivating the Tabat variety with 2P doses in Gezira. In addition, the Hakeka cultivar showed the highest increases in total flavonoid content, carotenoids, and antioxidant activities, particularly under the highest P level (2P). The findings highlight that P plays a critical role in enhancing sorghum’s nutritional and health-promoting qualities, which are essential for leveraging this staple crop for food and nutrition security strategies in semi-arid regions.

## Introduction

Sorghum (*Sorghum bicolor*) is a remarkably resilient crop well-adapted to harsh environmental conditions, making it a crucial player in ensuring future global food and feed security in the face of climate change threats [1]. It serves as a staple food source for millions in Sudan, where daily consumption ranges from 130 to 200 grams per person. Approximately 70% of the sorghum grains produced in Sudan are consumed at the household level, with the remainder used for sale and seed purposes [2]. The grain is utilized in the preparation of various traditional Sudanese food products, such as leavened bread (Kisra), stiff porridge (Aceda), thin fermented gruel (Nasha), and local beverages (Hulu-mur or Abreh) [3], as well as alcoholic beverages like (Merissa) and (Assaliya) [4]. Therefore, using sorghum in the Sudanese diet through indigenous methods and modern food products is essential to sustain market demand for the crop and ensure health and nutrition security.

Sorghum grains’ phenolic compounds, including phenolic acids, flavonoids, and tannins, contribute significantly to their antioxidant properties. Free radical scavengers such as these compounds reduce oxidative stress and protect cells from damage by reducing free radicals. For example, the 3-deoxy anthocyanidins in sorghum are especially effective antioxidants, contributing to the grain’s ability to resist environmental stressors [5]. This phenolic compound works as an antioxidant by donating electrons to neutralize free radicals, preventing lipid peroxidation and DNA and protein damage from oxidation. As well as chelating metal ions, they may create reactive oxygen species, which enhances their protective properties [6].

Nutritionally, these antioxidants contribute to sorghum’s potential to reduce the risk of chronic diseases such as cardiovascular disease and cancer. Thus, such functional foods containing high phenolic content and antioxidant activity are valuable, providing additional health benefits than essential nutrition [7].

Sorghum grain possesses attractive attributes highly relevant to modern food applications, particularly in chronic disease prevention [8]. The sorghum endosperm generally has a slower digesting starch profile than other cereal grains, which can contribute to slowed gastric emptying and potentially benefit satiety and weight management. The diverse array of phytochemicals present in sorghum, including tannins, phenolic acids, anthocyanins, carotenoids, phytosterols, and policosanols, alongside its proximate composition, vitamins, and minerals, are crucial for human health and nutrition, including improvements in glucose metabolism and insulin sensitivity, lipid metabolism, reduced fat accumulation, and decreased markers of oxidative stress and inflammation in recent human trials [1, 9, 10].

Despite its numerous health benefits, sorghum as a food ingredient remains underutilized compared to its potential [8]. In Sudan, sorghum production is predominantly (90%) rain-fed in the Central Clay Plains areas, with Phosphorus (P) deficiency contributing to declines in grain yield and quality [11]. While the total concentration of P in Sudanese soils is high, the concentration of inorganic phosphate (orthophosphate, H_2_PO_4_, abbreviated as Pi) in the soil solution, which is the primary source of P taken up by plants, is usually low, ranging from 1 to 10 μM. This low Pi availability limits the diffusion of Pi to plant roots and is primarily attributed to the low solubility of Pi minerals, Pi adsorption to soil particles, and the formation of organic P complexes [12]. Thus, to address this issue and ensure food security for the growing population, mineral fertilizers, including P fertilizers, have been widely applied to maintain high soil nutrient levels, favoring crop growth since the 1950s–1960s, a period known as the Green Revolution [13]. However, an appropriate P fertilization regime should be compatible with the growth pattern of sorghum. While numerous studies have investigated the effects of P fertilization on the phenolic compounds, fatty acid profiles, carotenoids, and tocochromanol content of various crops, there is a paucity of information regarding the impact of phosphate fertilization on phytochemical compositions and antioxidant activity in sorghum cultivars grown in the Central Clay Plains of Sudan. Abbas, Ahmad [14] reported that P fertilization had no significant effect on the phenolic compounds of cotton under water stress.

In contrast, Scagel and Lee [15] demonstrated that P fertilizer management altered the phenolic composition of basil plants, with increasing P enhancing the accumulation of Chicoric acid. Likewise, Ma, Zhang [16] demonstrated a significant increase in the total bound phenolic acid content in tested wheat cultivars in response to P fertilizer application. However, high P doses in the soybean field experiment increased protein and palmitic, oleic, and linolenic acid but decreased total lipid content [17]. Interestingly, the synergistic or antagonistic effect of P fertilization on the profiles of saturated and unsaturated fatty acids in maize grain is found to be dependent on the amount of P supplied, the genotypes of maize, and the soil environment [18]. Similarly, Lux, Schneider [18] showed that the profiles and concentrations of carotenoids and tocopherol of maize grains were unaffected by phosphate fertilization. They concluded that low phosphate availability did not impair corn grains’ biosynthesis of (poly)phenols, carotenoids, and tocochromanols. Therefore, this study aims to examine the influence of P application on phytochemicals, health-promoting compounds, and antioxidant activities in the grains of four Sudanese sorghum cultivars grown in two locations within the Central Clay Plains region.

## Materials and methods

### Plant materials, study area, experimental setup

This investigation included four sorghum cultivars with varying responses to Striga infestation. The susceptible varieties, *Tabat* and *P954063*, contrasted with the resistant varieties, *Hakika* (a hybrid of *P954063* and *SRN39*) and *Tetron*. Tabat, Hakika, and Tetron seeds were sourced from the sorghum breeding initiative at the Agricultural Research Corporation (ARC) in Sudan, and Professor Ejeta from Purdue University in West Lafayette, Indiana, USA, generously provided the P954063 seeds.

Field experiments were conducted in the summer of 2020 at two distinct research farms: White Nile state (WN) (13°10’ N, 32°40’ E) and Gezira state (14°24’ N, 33°29’ E). Both sites are characterized by clay soil classified as Typic Haplusterts.

A split-plot experimental design was implemented, with three replications totaling 36 plots. Each main plot was allocated to one of three P fertilizer levels (0P, 46 kg P2O5 ha-1, and 92 kg P2O5 ha-1), while the subplots (4 m by 3.2 m) housed the different sorghum cultivars.

The pre-planting procedures involved plowing, harrowing, and ridging the soil, and sorghum seeds were planted on the ridges with a spacing of 0.8 m between rows and 0.2 m within rows. Planting density was set at 100,000 plants per hectare, starting with five seeds per hole, later thinned to two seedlings. P was applied as triple super phosphate directly with the seeds, and urea (43 kg, N ha-1) was applied in two split doses, first 21 days post-sowing and again before panicle initiation. Irrigation intervals were adjusted based on rainfall, ranging between 10 to 12 days, and weeds were controlled manually using hand hoeing.

Five representative sorghum panicles per plot were selectively harvested from the inner rows to analyze for phytochemical and antioxidant properties at physiological maturity. However, these panicles were stored in individual cloth bags and sun-dried to less than 12% moisture content to prevent soil contact. Post-drying, grains were meticulously cleaned of any attached glumes, chaff, and debris. They were then transferred to new non-metallic envelopes and stored under cool conditions to minimize potential contamination from external particles.

This experiment was conducted in ARC research fields, and no permits were needed since it is part of this research project.

### Extraction procedure

The isolation of phenolic compounds, flavonoids, and antioxidants from sorghum grains was conducted based on a modified procedure by Talhaoui, Gómez-Caravaca [19]. Two grams of finely ground sorghum flour were suspended in 50 ml of methanol, maintaining a solid-to-liquid ratio of 1:25 (w/v). The mixture was agitated at 25°C for 24 hours on an orbital shaker (Skakapparat—50–1200 varv/min—Timer). This extraction was performed twice more on the residual material to ensure comprehensive extraction. The combined extracts were then concentrated under reduced pressure using a rotary evaporator (IKA rotationsindunstare, RV3 V, 0010003324) and stored for subsequent analysis.

### Quantification of total phenolic content

The extracts’ total phenolic concentration was determined using a modified Folin-Ciocalteu method [20]. Specifically, 20 μL of a 1:10 (w/v) dilution of the methanolic extract was mixed with 1.58 mL of distilled water and 100 μL of Folin-Ciocalteu reagent. After reacting for 8 minutes, 300 μL of sodium carbonate (Na2CO3) solution was added. The mixture was then vortexed for 10 minutes and allowed to stabilize in darkness at 20°C for 2 hours. A calibration curve using gallic acid standards was prepared to ensure accuracy, with absorbance measured at 765 nm using a spectrophotometer. Results were expressed in mg of gallic acid equivalents (GAE) per gram of dry weight.

### Determination of total flavonoid content

Total flavonoids were quantified using an adapted colorimetric technique described by Kim, Jeong [21]. One milliliter of the methanolic extract was mixed with 300 μL of 5% sodium nitrite (NaNO2) and 300 μL of 10% aluminum chloride (AlCl3). After 5 minutes at 25°C, 2 mL of 1 M sodium hydroxide (NaOH) was added, and the total volume was adjusted to 10 mL with distilled water. The solution was thoroughly mixed using a vortex. Absorbance was measured at 510 nm, and flavonoid content was calculated based on a catechin calibration curve, expressed as mg catechin equivalents (CE) per gram of dry weight.

### Antioxidant activity assays of sorghum grains

#### DPPH radical scavenging assay

In order to evaluate the antioxidant properties of sorghum grain extracts, the DPPH (1,1-diphenyl-2-picrylhydrazyl) assay was conducted following the procedure of Chang, Wu [22]. The assay involved mixing 0.9 mL of 50 mM Tris-HCl buffer (pH 7.4) with 0.1 mL of each extract or deionized water (as a control). The mixtures were incubated at ambient temperature for 30 minutes, followed by measurement of the absorbance at 517 nm using a spectrophotometer. Antioxidant activity was quantified regarding Trolox equivalents per gram of dry weight.

#### Total reducing power assay

The reducing power of the extracts was assessed using a modified protocol by Gülçin, Oktay [23] involving phosphate buffer and potassium ferricyanide. For this assay, methanolic extracts were prepared at 5 to 40 μg/mL concentrations. To 2.5 mL of each extract solution, 2.5 mL of 1% potassium ferricyanide and 2.5 mL of phosphate buffer (0.2 M, pH 6.6) were added. After heating at 50°C for 20 minutes, the reaction was quenched with 2.5 mL of 10% trichloroacetic acid. The mixture was centrifuged, and the supernatant was mixed with equal parts of distilled water and a few drops of 0.1% ferric chloride. Absorbance was read at 700 nm, and results were expressed in mg of ascorbic acid equivalents per gram of dry weight.

#### Ferric Reducing Antioxidant Power (FRAP) assay

The FRAP assay was carried out according to Oraiza [24] by mixing 0.5 mL of diluted methanol extract with 2 mL of a freshly prepared FRAP reagent (10 mmol/L TPTZ in 40 mmol/L HCl, 25 mL of 0.1 mol/L acetate buffer, pH 3.6, and 2.5 mL of 20 mmol/L FeCl3). This mixture was incubated at 37°C for 10 minutes in the dark, and absorbance was measured at 593 nm. Results were presented as micromoles of Trolox equivalents per gram of dry weight.

#### Hydrogen peroxide scavenging assay

The ability of the extracts to scavenge hydrogen peroxide was determined by mixing 1 mL of extract (1 mg/mL) with 3 mL of phosphate-buffered saline (PBS, pH 7.4) and 1 mL of 40 mM hydrogen peroxide according to the method of Jayaprakasha, Rao [25]. After a 10-minute incubation period, the absorbance of the reaction mixture was recorded at 230 nm. The scavenging activity was calculated as a percentage reduction in absorbance relative to the control.

#### ABTS radical scavenging activity

The ABTS radical scavenging activity was assessed using a pre-formed ABTS radical cation solution mixed with methanol to achieve an absorbance of 0.70 ± 0.05 at 734 nm, as Re, Pellegrini [26] described. An aliquot of 1 mL of this solution was added to 50 mL of each extract, and the reaction was allowed to proceed in the dark at room temperature for two hours. Absorbance was measured, and the antioxidant capacity was quantified as Trolox equivalents per gram of extract.

### Tannin determination

The quantification of tannins in the sorghum grain samples was performed using a modified vanillin-HCl spectrophotometric method [27]. Initially, 1 mL of methanol containing 1% hydrochloric acid (HCl) was combined with 5 mL of a vanillin reagent solution (comprising 4% HCl in methanol and 0.5 mL of vanillin). The mixture was incubated at 30°C, and the absorbance was measured at 500 nm after 20 minutes using a UV-visible spectrophotometer. Tannin concentrations were reported as catechin equivalents, providing a standardized measure of tannin content.

### Carotenoids determination

For carotenoid analysis, 2 grams of sorghum flour were extracted with 25 mL of cold acetone, followed by a separation process using 20 mL of petroleum ether [28]. The extract was then washed with 100 mL of distilled water to remove impurities. The final volume was adjusted back to 25 mL with petroleum ether. The absorbance of this prepared extract was measured at 450 nm. Carotenoid content was calculated using the formula: Total carotenoids (μg β-carotene g−1 DW) = (A × V × 104) / (E1% × P)

where A = absorbance at 450 nm, v = total extract volume, P = sample weight and E1% extinction coefficient of β-carotene in petroleum ether = 2592 (cuvette with 1 cm pathlength).

### Statistical analysis

Statistical tests were conducted to ensure data normality and homogeneity of variances using Shapiro-Wilks and Levene’s tests, respectively, with significance determined at P < 0.05. The influence of sorghum cultivars, P levels, and site locations on the bioactive compounds and antioxidant profiles was examined through a three-way ANOVA, utilizing SAS software version 9.1. P dependency (PD) was calculated based on the relative increase in phytochemicals in P-treated plants compared to untreated controls, expressed as a percentage. The PD is calculated as follows: (%) = [(grains phytochemical compositions of P treated plants- grains phytochemical compositions of non-P treated plant)/ the grains phytochemical compositions of P treated plants] X 100 [29]. The interaction analysis between the bioactive compounds, phosphorus levels, and locations was conducted in the Microsoft Excel Sheet. For P dependency, data were transformed by arcsine square root before analysis to satisfy the assumptions of normal distribution. Treatments with significant differences were analyzed using the Tukey HSD post hoc test. Multivariate analysis was conducted using HJ-Biplot PCA algorithms as described in the XLSTAT software [30]. Linear Partial Least Squares Regression test (PLS) was performed to validate and optimize the P application of four sorghum cultivars grown in two locations using the XLSTAT software [31].

### Ethics approval and consent to participate

All methods were carried out following relevant guidelines and regulations.

## Results

The results presented in (Table 1) illustrate the effect of different phosphate fertilizer levels on the TPC, TFC, tannin, and total carotenoid content of sorghum grain cultivars. The data demonstrates significant (p < 0.01) differences in TPC, TFC, total carotenoids, and tannin concentrations among the sorghum cultivars, phosphate fertilizer rates, and locations. Notably, applying P fertilizer resulted in a significant increase in all the measured phytochemicals compared to non-treated plants, with P fertilizer levels being the primary factor contributing to this effect.

**Table 1:** Total phenolics, total flavonoid, tannin, and total carotenoid contents of four sorghum cultivars in response to P fertilizers levels and grown at different locations.

	Sorghum bioactive compounds			
Cultivars	Total Phenolic (TPC)	Total Flavonoid (TFC)	Total Carotenoid	Tannin
Hakika	71.7b	68.3a	4.8a	4.5b
P954063	74.5a	64.7c	4.5b	4.8a
Tabat	59.1c	66.6b	2.5c	2.5c
Tetron	53.5d	55.7d	3.0d	2.5c
**P levels**				
0P	51.1c	57.2c	2.5c	3.2c
1P	66.4b	63.3b	3.9b	3.6b
2P	76.7a	71.0a	4.6a	3.9a
**Locations**				
Gezira	58.8b	65.4a	3.0b	4.0a
WN	70.6a	62.3b	4.5a	3.2b
**Three-Way ANOVA **				
Cultivars, C	4067.6***	956.2***	3905.7***	1128.1***
P levels, PL	8989.0***	1939.6***	4615.8***	100.8***
Locations, L	593.5***	307.5***	6830.8***	481.3***
C×PL	365.2***	58.6***	174.0***	2.4*
C×L	69.7***	2180.7***	2211.4***	80.5***
PL×L	150.2***	74.7***	707.4***	1.1ns
C×PL×L	105.6***	18.2***	216.6***	3.5**
SE±	1.8	1.3	0.2	0.15
CV%	1.3	1.2	2.1	4.4

Data were evaluated via three-way ANOVA, factors: four sorghum cultivars, Phosphorous fertilizer levels, and two locations. Identical letters indicate that values do not differ significantly at p < 0.05 according to Tukey HSD. Asterisks (*) indicate significantly influential factors as follows: ns, not significant

**, significant at p ≤ 0.01

***, significant at p ≤ 0.001 level. Each value represents the average of 3 replications.

For Total Phenolic Content (TPC), the control group (0P) recorded 51.1 mg GAE/g, whereas the treatments showed substantial increases, with 1P reaching 66.4 mg GAE/g (a 29.9% increase) and 2P at 76.7 mg GAE/g (a 50.1% increase). Similarly, Total Flavonoid Content (TFC) increased from 57.2 mg CE/g in the control to 63.3 mg CE/g (a 10.7% increase) with 1P and 71.0 mg CE/g (a 24.1% increase) with 2P.

Total Carotenoid Content also significantly rose, starting from 2.5 mg/g in the control, with 3.9 mg/g (a 56% increase) under 1P and 4.6 mg/g (an 84% increase) under 2P. Tannin Content followed a similar trend, increasing from 3.2 mg/g in control to 3.6 mg/g (a 12.5% increase) under 1P and 3.9 mg/g (a 21.9% increase) under 2P.

The sorghum plants grown in Gezira state showed significantly higher TFC and tannin concentrations than sorghum grown in White Nile, except for TPC and carotenoid concentrations, which showed the reverse trend. All three factors significantly interacted, except for tannin concentration, for which the interaction between P fertilizer levels and locations was insignificant.

Regarding the tested sorghum cultivars, cultivar P954063 had significantly higher (p < 0.01) TPC and tannin concentrations than the other three cultivars. The cultivar Hakakia had significantly higher (p < 0.05) TFC and total carotenoid concentrations compared to the other three cultivars. In contrast, the cultivar Tetron demonstrated the lowest TPC (53.5 GAE g-1 D.W.), TFC (55.7 CE g-1 D.W.), total carotenoids (3.0), and tannin (2.5 CE g-1 D.W.) concentrations. Notably, no significant differences in grain tannin concentrations between Tabat and Tetron cultivars were observed.

Table 2 evaluates antioxidant activity in sorghum grains using DPPH, FRAP, TRP, H2O2, and ABTS assays. The results showed a significant difference (p < 0.05) among sorghum cultivars, phosphate application, locations, and their possible interactions. The highest in vitro antioxidant activities were observed in sorghum plants treated with phosphate fertilizer compared to the non-treated, except for H2O2, whose grain concentration significantly (p < 0.01) decreased with phosphate application compared to the non-P treated control. Moreover, increasing P levels gradually undermined the Effect of P on the grains’ H2O2 concentrations. DPPH rose from 2.3 mg TE/g in the control to 2.9 mg TE/g (a 26.1% increase) with 1P and 3.1 mg TE/g (a 34.8% increase) with 2P. FRAP showed similar improvements, increasing from 4.3 mg TE/g in the control to 5.2 mg TE/g (a 20.9% increase) with 1P and 6.0 mg TE/g (a 39.5% increase) with 2P. Total Reducing Power (TRP) exhibited the most dramatic change, surging from 1.9 mg AAE/g in the control to 7.0 mg AAE/g (a 268% increase) with 1P and 9.3 mg AAE/g (a 389.5% increase) with 2P. Interestingly, Hydrogen Peroxide Activity decreased slightly, from 80.0% in the control to 78.2% (a 2.3% decrease) with 1P and further to 76.9% (a 3.9% decrease) with 2P. Lastly, ABTS Radical Scavenging Activity showed minimal changes, moving from 3.32 mg TE/g in the control to 3.33 mg TE/g (a 0.3% increase) with 1P and 3.34 mg TE/g (a 0.6% increase) with 2P.

**Table 2:** Free radical scavenging DPPH (mg/g dry weight), ferric reducing antioxidant power FRAP (mg/g dry weight), total reducing power (AAE/g sample), hydrogen peroxide free radical scavenging activity and ABTS radical scavenging activity (milligrams of Trolox equivalents (TE) per gram) of four sorghum cultivars in response to phosphorus fertilization at different locations.

	*In-vitro* antioxidant activities				
Cultivars	DPPH (Trolox (ET mg/100g)	FRAP (Trolox mg/g)	TRP ascorbic acid (mg/g)	Hydrogen Peroxide activity (%)	ABTS activity
Hakika	4.0a	5.8a	7.3a	76.6c	3.35a
P954063	3.0b	5.6b	7.2a	77.4b	3.35a
Tabat	2.0d	4.7c	5.5b	84.9a	3.31b
Tetron	2.1c	4.7c	4.2c	74.6d	3.31b
**P levels**					
0P	2.3c	4.3c	1.9c	80.0a	3.32c
1P	2.9b	5.2b	7.0b	78.2b	3.33b
2P	3.1a	6.0a	9.3a	76.9c	3.34a
Locations					
Gezira	1.7b	4.7b	4.5b	77.7a	3.32b
WN	3.9a	5.7a	7.6a	79.1b	3.34a
**Three-Way ANOVA **					
Cultivars, C	11424.8***	3685.8***	289.7***	3098.4***	816.1***
P levels, PL	2373.6***	10251.9***	2587.0***	504.8***	268.3***
Locations, L	59652.6***	11670.1***	1281.5***	289.1***	779.9***
C×PL	231.3***	76.8***	15.7***	93.0**	39.2**
C×L	229.8***	840.8***	19.1***	61.0**	4.3*
PL×L	50.5***	20.0***	547.5***	79.1**	10.1**
C×PL×L	127.0***	91.8***	5.1***	45.3**	7.6**
SE±	0.17	0.12	0.47	0.53	0.01
CV%	1.4	0.79	6.0	0.56	0.23

Data were evaluated via three-way ANOVA, factors: four sorghum cultivars, Phosphorous fertilizer levels, and two locations. Identical letters indicate that values do not differ significantly at p < 0.05 according to Tukey HSD. Asterisks (*) indicate significantly influential factors as follows: ns, not significant

**, significant at p ≤ 0.01

***, significant at p ≤ 0.001 level. Each value represents the average of 3 replications.

Regarding the locations, sorghum plants grown in the White Nile had significantly (p < 0.01) greater DPPH, FRAP, TRP, and ABTS than those grown in Gezira. Meanwhile, sorghum plants grown in Gezira exhibited the highest grain H_2_O_2_ concentration.

The cultivar Hakika had the highest DPPH, FRAP, TRP, and ABTS concentrations among the tested sorghum cultivars (Table 2). Cultivars Hakika and P954063 did not differ significantly in TRP and ABTS concentrations. However, cultivars Tabat and Tetron had the lowest FRAP and ABTS concentrations, significantly different from the cultivars Hakika and P954063 (p < 0.01). Cultivar Tabat showed the highest H2O2 grain concentration, significantly different from the tested three sorghum cultivars (p < 0.01).

Tables 3 and 4, respectively, analyze the P dependency of sorghum on phytochemical compounds and antioxidant activity.

**Table 3:** Phosphorus dependency of sorghum bioactive compounds as influenced by sorghum cultivars, P fertilizer levels, location and their interactions.

		Sorghum bioactive compounds		
Cultivars	TPC	TFC	Total carotenoids	Tannin
Hakika	38.3a	15.6b	43.2z	6.1c
P954063	21.4b	16.4ab	31.8b	10.0bc
Tabat	38.3a	7.2c	47.6z	24.4a
Tetron	23.9b	19.2a	28.6b	16.8ab
**P levels**				
1P	22.0b	9.8b	32.4b	10.3b
2P	38.9a	19.4a	43.2a	18.4a
**Locations**				
Gezira	28.9b	15.3a	31.2b	13.6a
WN	32.0a	13.9b	44.3a	15.0a
**Three-Way ANOVA **				
Cultivars, C	370.7***	92.4***	124.1***	36.5***
P levels, PL	1268.3***	317.7***	176.9***	33.8***
Locations, L	43.9***	6.3*	259.7***	2.5^NS^
C×PL	176.9***	9.1*	2.9^NS^	0.1^NS^
C×L	198.0***	7.6*	156.1***	1.7^NS^
PL×L	12. 7**	26.1**	0.4^NS^	0.6^NS^
C×PL×L	17.9**	4.0*	9.8**	0.8
SE±	2.1	1.1	2.2	1.35
CV%	5.4	12.8	.5	7.5

Data were evaluated via three-way ANOVA, factors: four sorghum cultivars, Phosphorous fertilizer levels and two locations. Identical letters indicate that values do not differ significantly at p < 0.05 according to Tukey HSD. Asterisks (*) indicate significantly influential factors as follows: ns, not significant

**, significant at p ≤ 0.01

***, significant at p ≤ 0.001 level.

**Table 4:** Phosphorus dependency of in vitro antioxidant activity assays as influence by sorghum cultivars and P fertilizer levels, location and their interactions.

		*In-vitro* antioxidant activities		
Cultivars	DPPH	FRAP	TRP	ABTS
Hakika	2.5c	25.0a	65.8b	0.8a
P954063	22.9b	21.5bc	75.7b	0.9a
Tabat	49.1a	21.3c	103.2a	0.2b
Tetron	51.4a	22.6b	114.8a	0.2b
**P levels**				
1P	30.0b	17.1b	67.0b	0.4b
2P	32.9a	28.2a	112.7a	0.7a
**Locations**				
Gezira	47.7a	24.5a	82.2b	0.4b
WN	15.3b	20.7b	97.5a	0.6a
**Three-Way ANOVA **				
Cultivars, C	1332.0***	5.1**	69.1***	179.1***
P levels, PL	20.7**	194.0***	273.2***	107.3***
Location, L	2578.0***	229.2***	31.0***	16.4**
C×PL	38.9**	27.2**	18.5**	5.1**
C×L	768.1***	156.2***	17.2**	16.1**
PL×L	26.2**	1.6^NS^	67.2 ***	8.5**
C×PL×L	32.5**	54.9**	5.3*	9.8**
SE±	4.54	1.1	5.5	0.04
CV%	7.0	3.9	10.6	9.8

Data were evaluated via three-way ANOVA, factors: four sorghum cultivars, Phosphorous fertilizer levels and two locations. Identical letters indicate that values do not differ significantly at p < 0.05 according to Tukey HSD. Asterisks (*) indicate significantly influential factors as follows: ns, not significant

**, significant at p ≤ 0.01

***, significant at p ≤ 0.001 level.

Table 3 illustrates a significant P dependency for TPC (total phenolic content), TFC (total flavonoid content), and total carotenoid differences among sorghum cultivars, P levels, and locations. The interaction between these three factors remained significantly different for TPC and TFC (P < 0.01). However, there was no significant P dependency for total carotenoid interactions between sorghum cultivars and P levels and between P levels and locations. Tannin concentration did not show a significant P dependency effect for locations or possible factorial interactions.

Phosphate application notably enhanced the P dependency for all tested bioactive compounds (P < 0.01), and the enhancement was dependent on P fertilizer levels, as shown in Table 3. The P dependency was significantly higher in sorghum plants grown in White Nile compared to the Gezira for TPC and total carotenoid contents. At the same time, the situation was reversed for TPC and tannin.

For in vitro antioxidant activity, prominent influences of sorghum cultivars, P fertilizer levels, locations, and their interactions were detected (except for FRAP, where the interaction effect between P fertilizer levels and locations was not statistically different), as displayed in Table 4. Increasing P fertilizer levels resulted in a P dependency for all tested antioxidant activities. Among the locations, sorghum plants grown in the Gezira showed significantly higher P dependency (P < 0.01) for DPPH and FRAP concentrations compared to those grown in WN. However, the situation was reversed for TRP and ABTS concentrations.

**Fig 1:**
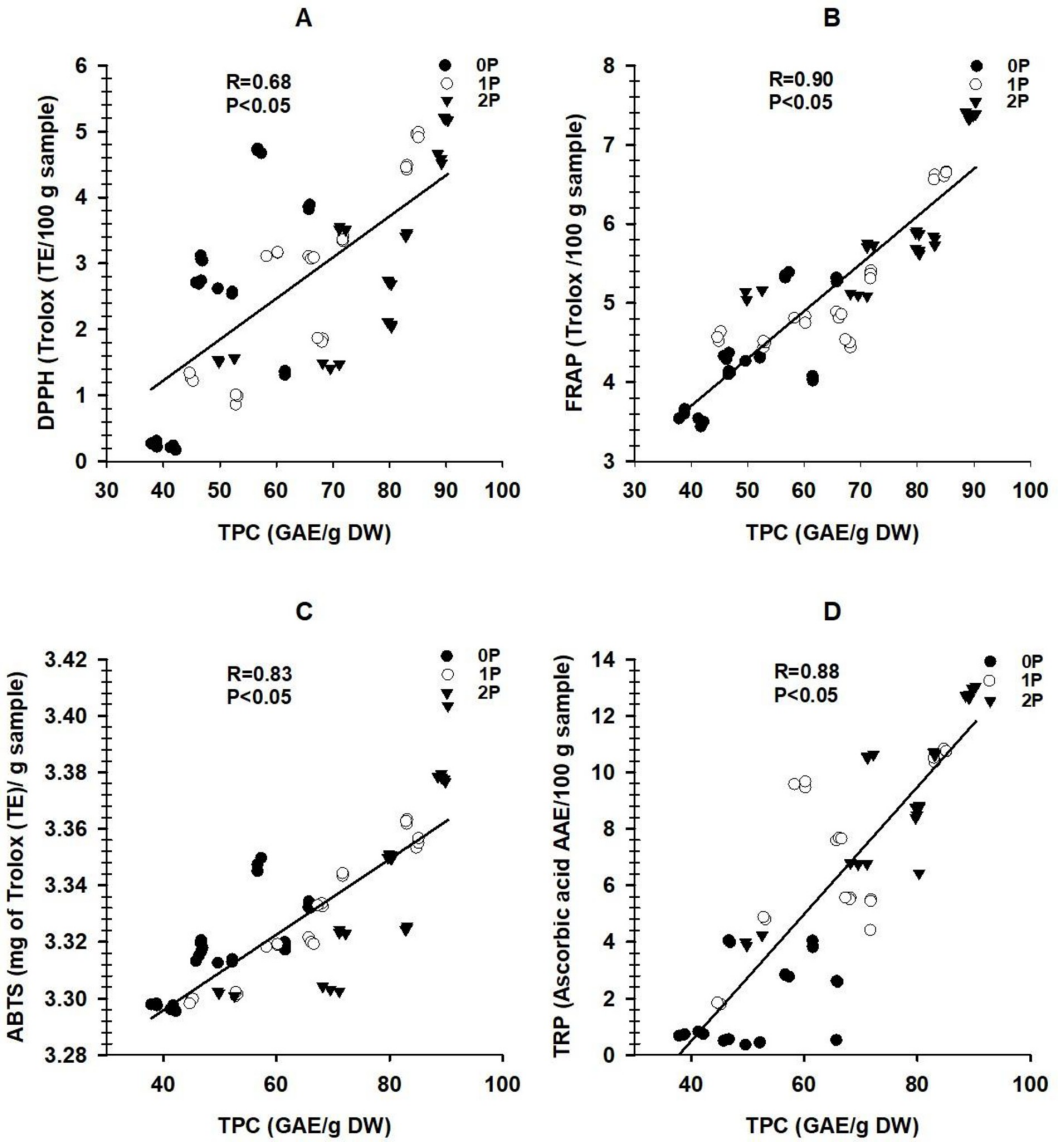
(A) Relationships between total phenolic contents and free radical scavenging DPPH, (B) Total phenolic contents and ferric reducing antioxidant power, (C) Total phenolic contents and ABTS radical scavenging activity, (D) Total phenolic contents and total reducing power.

**Fig 2:**
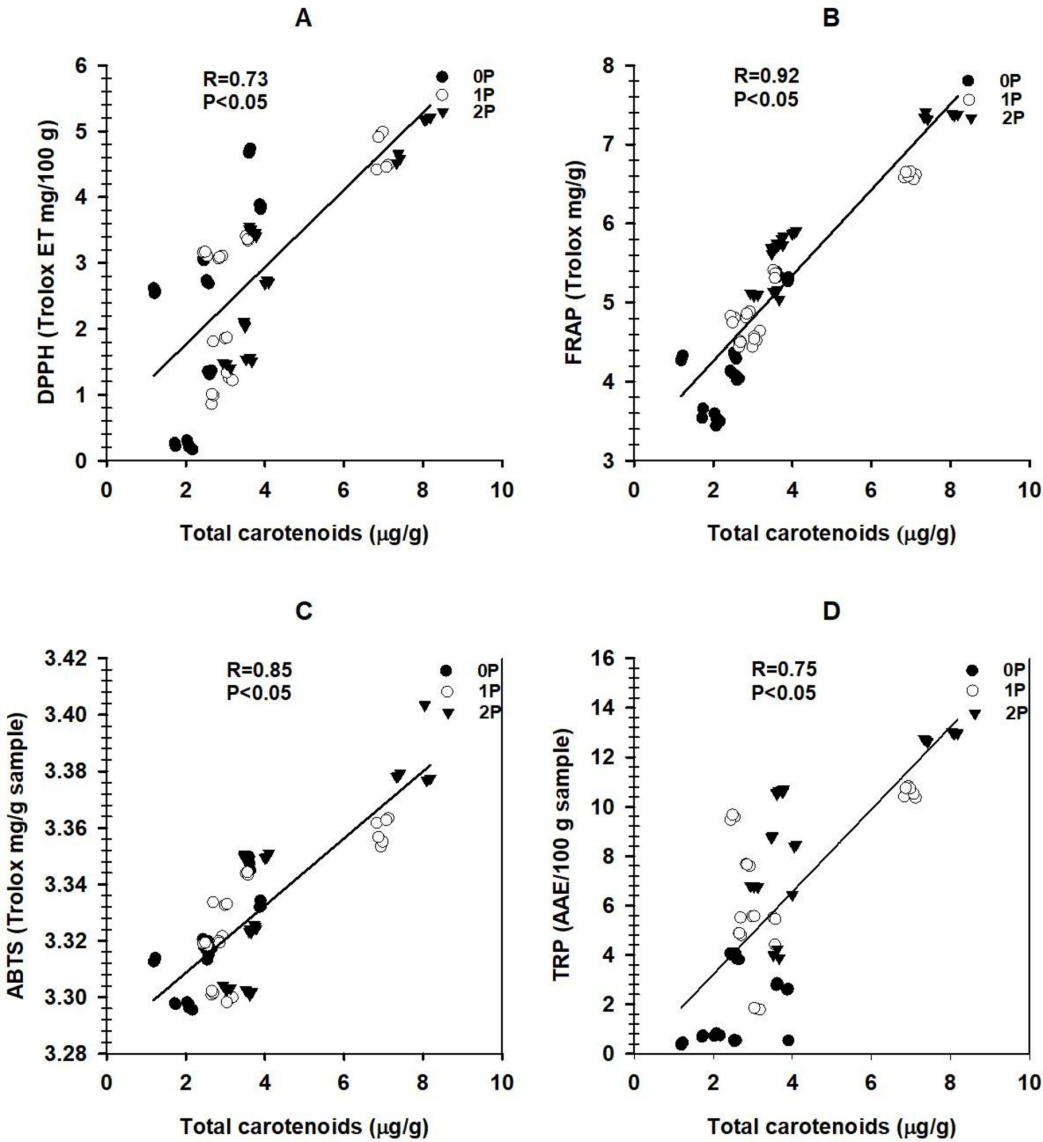
(A) Relationships between total carotenoids content and free radical scavenging DPPH, (B) Total carotenoids content versus ferric reducing antioxidant power, (C) Total carotenoids content versus ABTS radical scavenging activity, (D) Total carotenoids content and total reducing power.

Significant correlations were found between total phenolic concentrations and all antioxidant assays: DPPH (r = 0.68), FRAP (r = 0.90), ABTS (r = 0.83), and TRP (r = 0.88) (Fig 1A–1D). Total carotenoids also showed strong correlations with antioxidant activities: DPPH (r = 0.73), FRAP (r = 0.93), ABTS (r = 0.85), and TRP (r = 0.75) (Fig 2A–2D).

**Fig 3:**
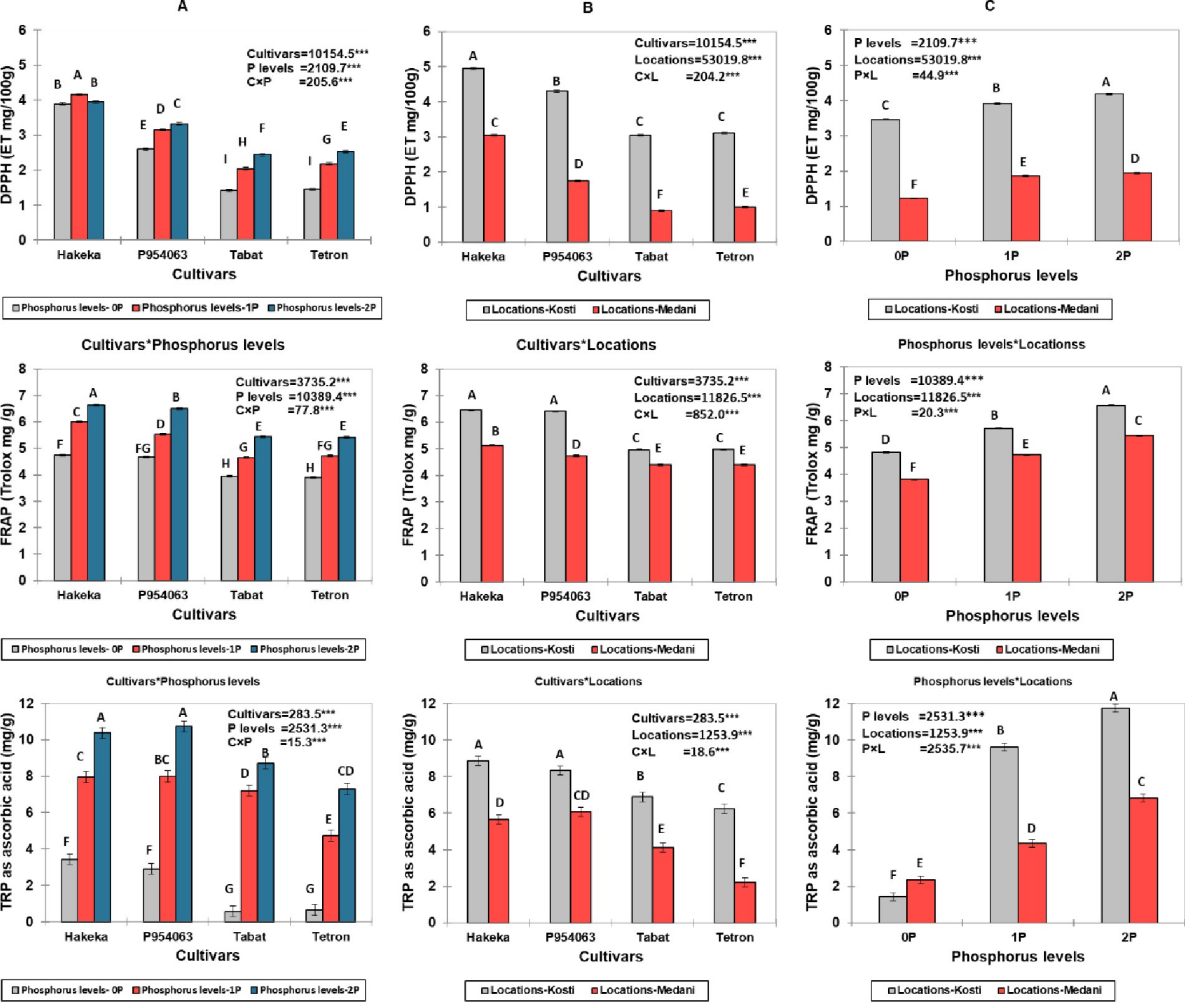
Interactions between cultivars, phosphorus levels, and locations for three antioxidant parameters: DPPH radical scavenging activity, ferric reducing antioxidant power (FRAP), and total phenolic content (TPC). (A) Interactions between cultivars and phosphorus levels, (B) Interactions between cultivars and locations, (C) Interactions between phosphorus levels and locations.

Significant interactions were observed between cultivars, phosphorus levels, and locations for DPPH activity, FRAP values, and TPC (p<0.001 for all interactions) (Fig 3A–3C). Among the cultivars, Hakeka exhibited the highest DPPH activity, particularly at the 1P and 2P phosphorus levels. Location significantly influenced DPPH activity, with Kosti generally presenting higher values than Medani across all cultivars and phosphorus levels. For FRAP values, significant interactions were also found between cultivars, phosphorus levels, and locations (p<0.001 for all interactions). The Hakeka and P954063 cultivars recorded the highest FRAP values, notably at the 2P phosphorus level. The Kosti location consistently exhibited higher FRAP values than Medani across all cultivars and phosphorus treatments. Similarly, TPC results showed significant interactions among cultivars, phosphorus levels, and locations (p<0.001 for all interactions). The P954063 and Hakeka cultivars demonstrated the highest TPC, particularly at the 2P phosphorus level. Again, the Kosti location generally yielded higher TPC values than Medani across all treatments. Thus, increasing phosphorus levels from 0P to 2P generally enhanced antioxidant activity and phenolic content, with the most pronounced effects observed in the Kosti location. The cultivars Hakeka and P954063 consistently outperformed Tabat and Tetron in antioxidant properties under various phosphorus treatments and locations.

**Fig 4:**
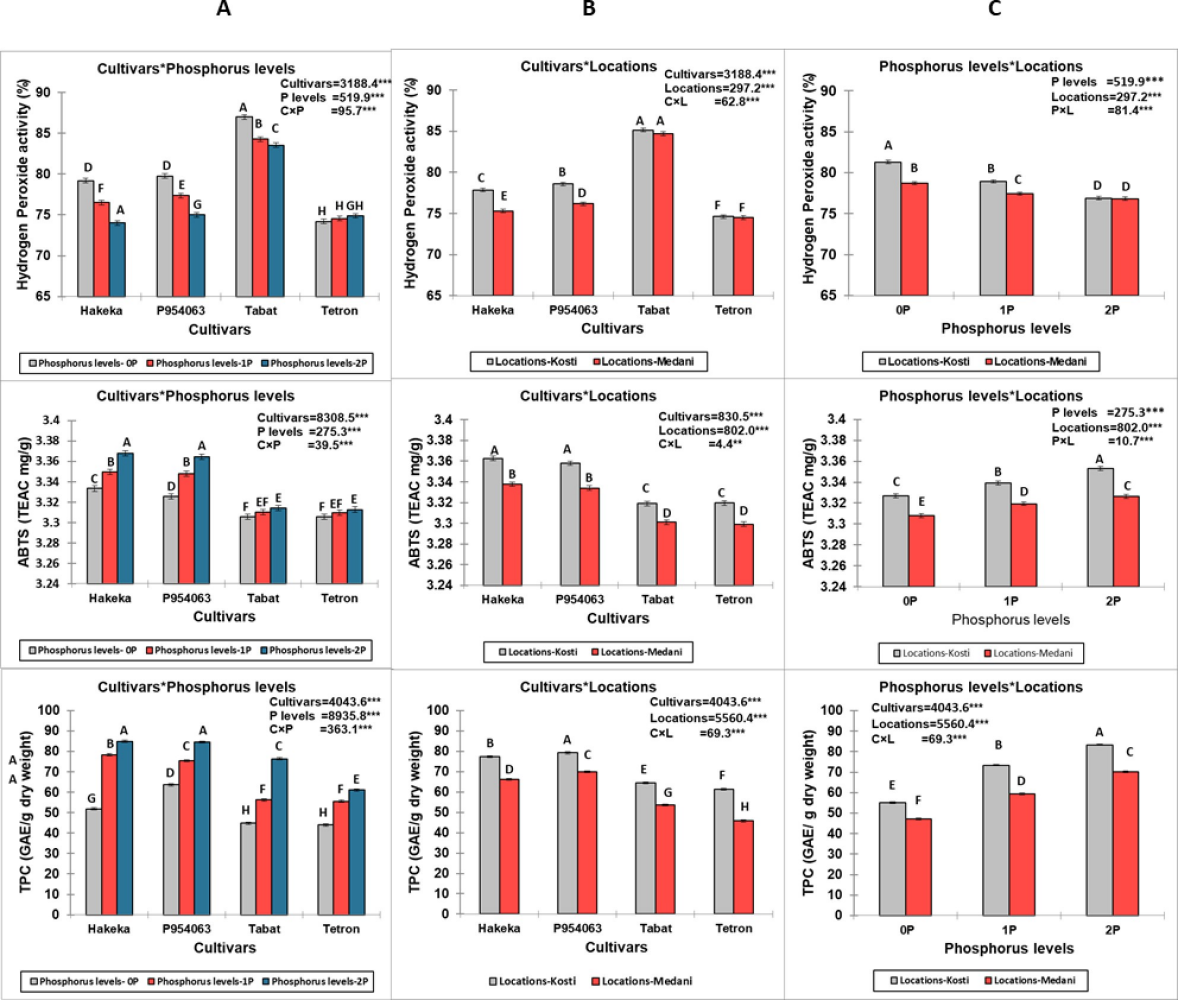
Interactions between cultivars, phosphorus levels, and locations for hydrogen peroxide activity, ABTS, and TPC. (A) Interactions between cultivars, phosphorus levels, (B) Interactions between cultivars and locations, (C) Interactions between phosphorus levels and locations.

Significant interactions were observed for hydrogen peroxide activity, ABTS, and TPC among cultivars, phosphorus levels, and locations (p<0.001 for all interactions) (Fig 4A–4C). The Hakeka and P954063 cultivars consistently exhibited the highest values across these parameters, particularly at the 2P phosphorus level. Location also played a crucial role, with Kosti showing consistently higher hydrogen peroxide activity, ABTS TEAC values, and TPC than Medani, regardless of phosphorus level. This data suggests that increasing phosphorus levels enhances antioxidant activities and phenolic content, with the most pronounced effects observed in the Kosti location, especially for the Hakeka and P954063 cultivars.

**Fig 5:**
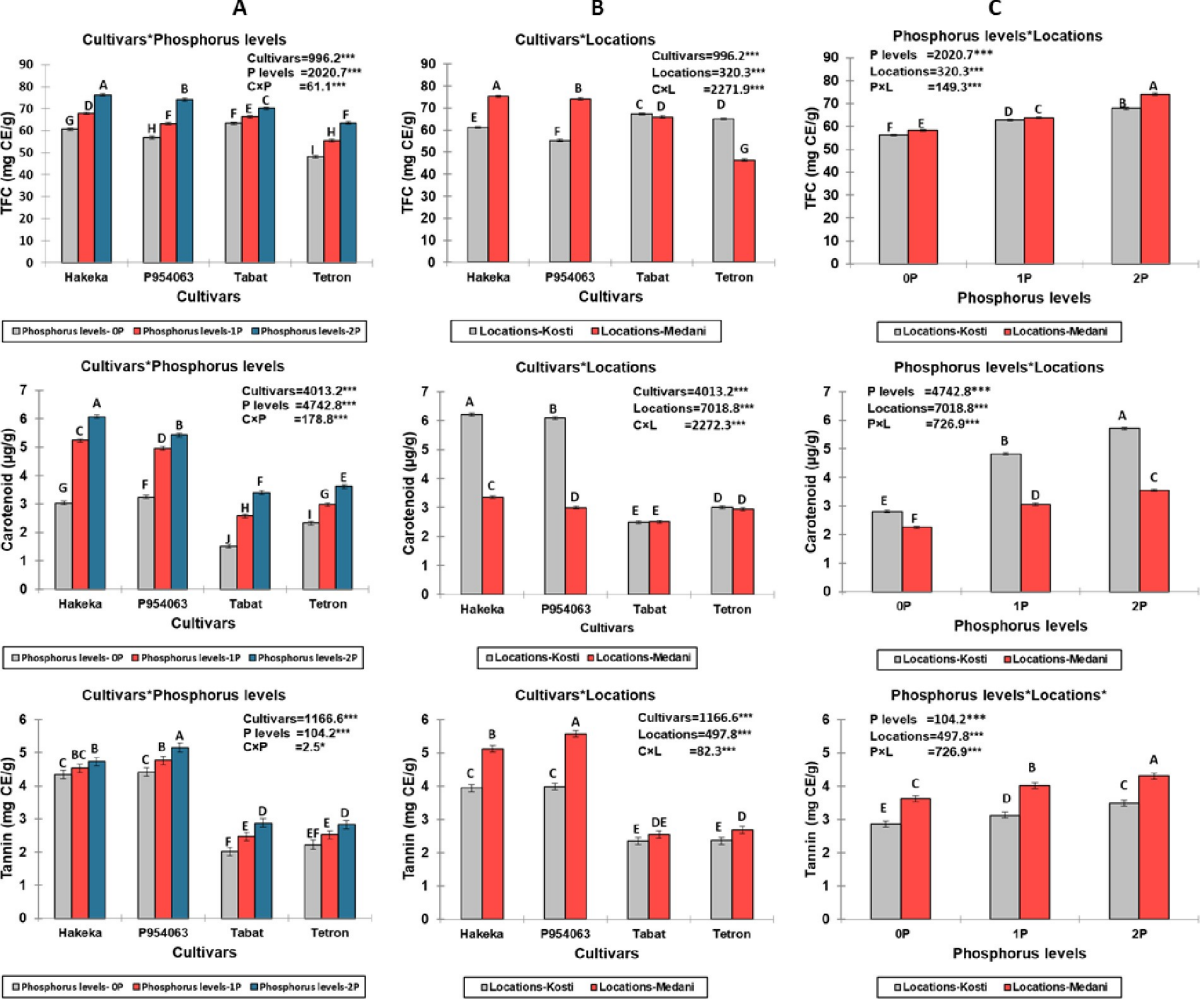
Interactions between cultivars, phosphorus levels, and locations for total flavonoid content (TFC), carotenoid content, and tannin content. (A) Interactions between cultivars and phosphorus levels, (B) Interactions between cultivars and locations, (C) Interactions between phosphorus levels and locations.

Significant interactions were observed for total flavonoid content (TFC), carotenoid content, and tannin content among cultivars, phosphorus levels, and locations (p<0.001 for all interactions) (Fig 5A–5C). The Hakeka cultivar consistently exhibited the highest values across these parameters, particularly at the 2P phosphorus level. The location also played a crucial role but with varying effects depending on the measured compound. For TFC, Medani showed consistently higher values than Kosti, regardless of phosphorus level or cultivar. In contrast, Kosti exhibited higher values for carotenoid content than Medani across all conditions. Tannin content was generally higher in Medani than in Kosti, except for Hakeka at 0P, where Kosti showed a higher value. P954063 typically showed the second-highest values for all three compounds, followed by Tabat and Tetron. The effect of increasing phosphorus levels was generally positive for all measured compounds, with the most pronounced increases observed between 0P and 1P levels. These results suggest that increasing phosphorus levels enhances flavonoid, carotenoid, and tannin content, with the effect varying by cultivar and location. The most pronounced effects were typically observed in the Hakeka cultivar, while the optimal location varied depending on the specific compound being measured.

**Fig 6:**
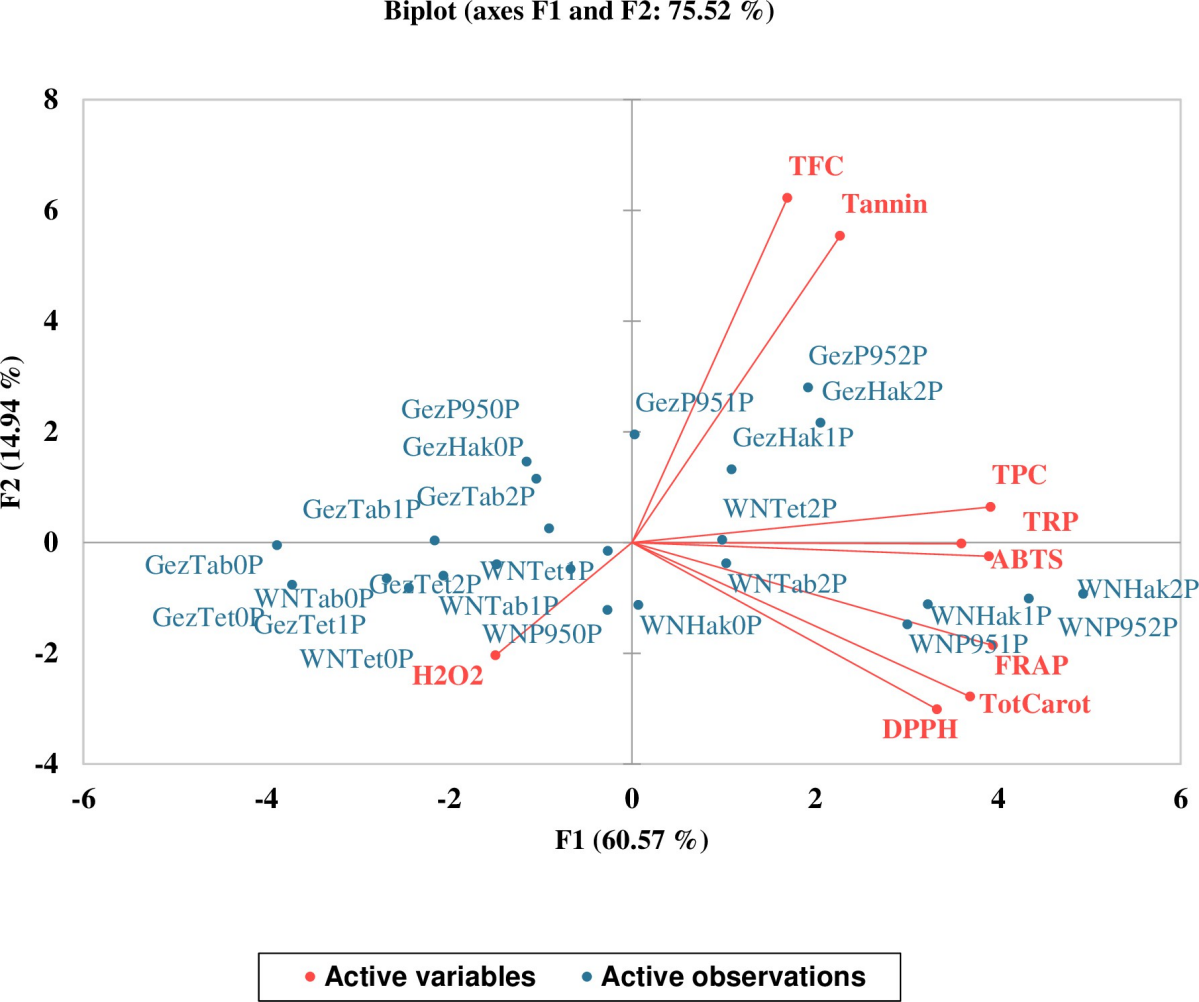
Principal component (PC) scores for the experimental variables determined in grains of four sorghum cultivars treated with different phosphorus fertilizer levels. The percentage values in parentheses indicate the variation explained by each PC. The plot shows the distribution of the experimental individuals according to the PCs and grouped according to phosphorus levels.

Based on a PCA (Principal Component Analysis), the samples from P-treated sorghum plants (1P & 2P) and non-treated P sorghum plants (0P) were separated, indicating differences in both P levels and locations. However, a slight overlap between the two P levels was observed (Fig 6).

The first two principal components (PC1 and PC2) accounted for much of the total variation, explaining 60.6% and 14.9%, respectively. The eigenvectors corresponding to PC1 included DPPH, FRAP, TRP, ABTS, TPC, and total carotenoid contents, while the eigenvectors corresponding to PC2 included TFC and tannin. These eigenvectors contributed to the differentiation in both P levels and locations. Notably, there was a strong positive correlation between the measured parameters and P-treated sorghum plants, except for hydrogen peroxide (H2O2). These findings indicate that applying phosphate fertilizer enhanced the synthesis of sorghum’s health-promoting phytochemicals and antioxidant activities.

**Fig 7:**
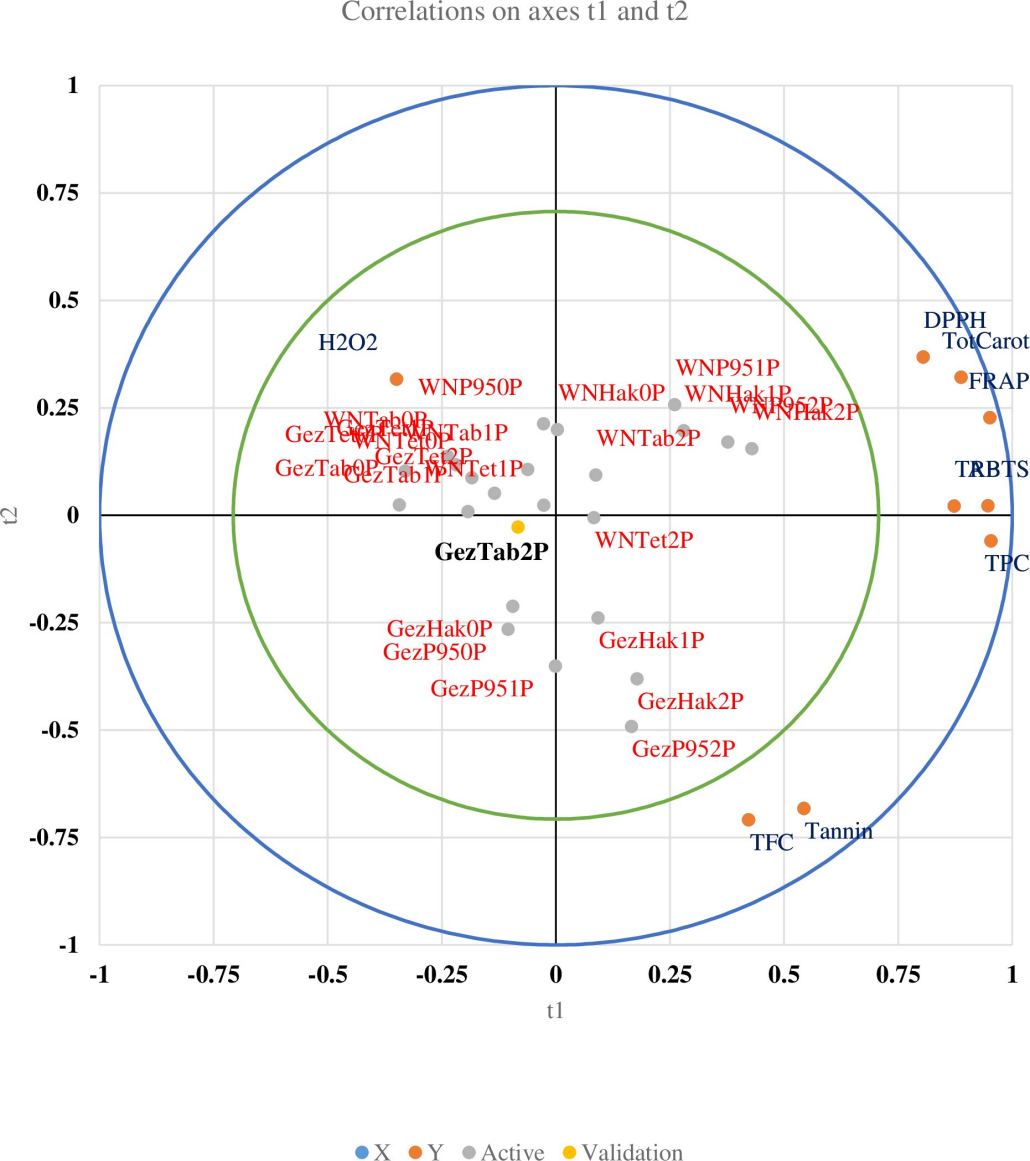
Partial Least Squares regression analysis (PLS) for the experimental variables determined in grains of four sorghum cultivars treated with different phosphorus fertilizer levels in two locations.

PLS (Partial Least Squares) analysis was conducted to examine the interactive effects of phosphate fertilization and non-treated control on the measured parameters of sorghum grain cultivars grown in the Gazira and White Nile locations (Fig 7). The results showed that, regardless of the P doses, phosphate fertilization had a positive validation score for most of the studied parameters, except for H_2_O_2_, across all sorghum cultivars and locations. Moreover, the PLS analysis identified that the phosphate fertilization at the P2 level for the sorghum cultivar Tabat in the Gezira location (GezTab2P) was the most valid, suggesting its potential to produce high-quality grains.

These findings highlight the significance of phosphate fertilizer application in enhancing the production of health-promoting phytochemicals and antioxidant activities in sorghum, as demonstrated by the PCA and PLS analyses.

## Discussion

The present study investigated the effects of P fertilization on the phytochemical composition and antioxidant activities of sorghum grains from four cultivars grown in two locations in the Central Clay Plains of Sudan. P is an essential macronutrient crucial in various plant metabolic processes, including the biosynthesis of secondary metabolites and regulating antioxidant defense systems [32]. Understanding the impact of P availability on these bioactive compounds and their associated antioxidant capacities is vital for optimizing the nutritional quality and potential health benefits of sorghum as a food crop.

Our results demonstrate a notable increase in the concentrations of health-promoting phytochemical compounds in sorghum grains, including total phenolics, flavonoids, carotenoids, and tannins in response to P application. However, this positive influence of phosphate application can be explained from four perspectives. Firstly, P application significantly increases the activity of enzymes and transcription factors involved in the biosynthetic pathways of the studied health-promoting phytochemical compounds [33]. For instance, Khan, Prithiviraj [34] found that P application enhanced the activity of phenylalanine ammonia-lyase (PAL), a key enzyme in the phenylpropanoid pathway, leading to increased phenolic compound accumulation in soybean. Consequently, plants can produce and accumulate higher concentrations of bioactive compounds, such as phenolics, flavonoids, carotenoids, and tannins, in their grains [35].

Secondly, P availability in the soil can regulate the activity of transcription factors that control the expression of genes related to secondary metabolism [36]. A study by Zhang, Jiang [37] reported that P deficiency downregulated the expression of genes involved in the biosynthesis of flavonoids and carotenoids in tomato, leading to reduced accumulation of these compounds.

Thirdly, P availability can ensure the accumulation of bioactive compounds by enhancing the antioxidant defense system in plants through the activation of antioxidant enzymes, such as superoxide dismutase (SOD), catalase (CAT), and peroxidase (POD), which help scavenge reactive oxygen species (ROS). According to Gill and Tuteja [38], ROS are known to negatively affect plant growth and subsequently protect the integrity of plant cells, ensuring the accumulation of bioactive compounds in seeds. Arora, Jain [39] reported that P deficiency led to increased ROS production and oxidative stress in wheat plants, which could impair the biosynthesis and accumulation of antioxidant compounds.

Lastly, it could be hypothesized that phosphate application alters metabolic processes by interacting with other nutrients in the soil, creating a balanced nutrient environment, which results in proper plant growth and metabolism, potentially increasing the concentrations of bioactive compounds [40, 41]. In addition, Alam, Carpenter-Boggs [42] observed that balanced P and nitrogen nutrition enhanced the accumulation of phenolic compounds and antioxidant activities in pea. Therefore, proper P management strategies are essential to maximize the accumulation of bioactive compounds in sorghum grains, at least in the Central Clay Plain of Sudan.

On the other hand, in this study, phosphate application prominently increased the antioxidant activities of DPPH, FRAP, TRP, and ABTS in sorghum grains (Table 2). These findings can be attributed to the enhancement of antioxidant enzyme activities, namely superoxide dismutase (SOD), catalase (CAT), peroxidase (POD), and ascorbate peroxidase (APX), which play a crucial role in neutralizing reactive oxygen species (ROS) and protecting cells from oxidative damage [38]. P availability can influence the biosynthesis and accumulation of non-enzymatic antioxidant compounds, such as phenolics, flavonoids, carotenoids, and other phytochemicals, contributing to the overall antioxidant capacity of grains [43]. Chrysargyris, Petropoulos [44] demonstrated that P application increased the concentrations of phenolic compounds and flavonoids in (*Matricaria chamomilla* L), leading to enhanced antioxidant activities.

The synthesis of phytochemicals in plants is closely tied to phosphorus (P) pathways, as phosphorus is essential for energy transfer and the regulation of biosynthetic processes. Phosphorus, a key component of ATP, powers enzymatic reactions critical for the biosynthesis of secondary metabolites like phenolics and flavonoids, which are responsible for plants’ antioxidant activity. Plants manage phosphorus acquisition and homeostasis through a complex network involving phosphate transporters and Pi starvation-induced genes, directly influencing phytochemical production [45]. Phosphorus limitation induces the expression of genes that enhance the production of specific phytochemicals, which function as protective agents against oxidative stress, thus boosting the plant’s antioxidant capacity [46].

Moreover, P availability influences the electron transfer capacity of plant tissues, which is crucial for antioxidant activities [47]. Santos-Sánchez, Salas-Coronado [48] concluded that the electron transfer capacity is associated with the ability of compounds to donate or accept electrons, thus neutralizing free radicals and reducing oxidative stress. Ultimately, P availability in the soil is closely associated with the efficient functioning of antioxidant defense systems, leading to higher *in-vitro* antioxidant activities in sorghum grains.

However, a reverse reaction was encountered for hydrogen peroxide (H_2_O_2_), as phosphate application significantly reduced the H_2_O_2_ scavenging capacity, and the reduction was dependent on P levels. This finding could be attributed to the stabilization of plant cell membranes and the balancing of redox, which significantly reduced ROS production. However, these factors significantly decrease H_2_O_2_ concentrations in plant tissues and grains [49].

Our results indicated that P dependency for all tested bioactive compounds and antioxidant activities was most significant in the Gezira location with poor soil compared to the White Nile (WN) location. It could be hypothesized that the White Nile location contains sufficient soil P to supply sorghum plants until maturity; therefore, no effect of P fertilization is observed. Unsurprisingly, P fertilization has been reported to be effective under P-deficient soil conditions, as opposed to conditions of sufficient P supply [50], which could justify the reduction in P dependency observed in the WN soil.

In addition, the study shows strong positive correlations between total phenolics, carotenoids, and measured antioxidant activities (Figs 1 and 2). These positive correlations are anticipated, as total phenolics and carotenoids often work synergistically, enhancing each other’s antioxidant activities. They may have complementary mechanisms of action and can scavenge a broader range of free radicals and reactive species. Consequently, the combined presence of both compounds in higher concentrations can produce more substantial antioxidant effects [51], indicating positive correlations with measured antioxidant activities in sorghum grains. Eventually, total phenolics and carotenoids possess unique aromatic rings and hydroxyl, enabling them to scavenge free radicals and donate electrons [52]. Notably, the conjugated double bonds of carotenoids are reported to neutralize reactive species in plants [53] and enhance the grains’ antioxidant activities.

The partial least squares (PLS) analysis for locations, P doses, and their interaction with the measured parameters indicates that the cultivar Tabat tested in Gezira was the most suitable for cultivation (Fig 4). Tabat in Gezira with a 2P dose significantly improved the health-promoting metabolites in sorghum-derived food. Therefore, cultivating Tabat in Gezira is recommended for this purpose.

## Conclusions

This study demonstrates that phosphorus (P) fertilization significantly enhances sorghum grains’ phytochemical content and antioxidant activities, with notable variations across different cultivars and geographic locations. Specifically, the Hakeka cultivar showed the highest increases in total flavonoid content, carotenoids, and antioxidant activities, particularly under the highest P level (2P). Also, the cultivar Tabat in Gezira with a 2P was validated as significantly improving the health-promoting metabolites in sorghum. The findings underscore the importance of tailored P fertilization strategies that consider both the genetic characteristics of sorghum cultivars and local soil conditions to optimize the crop’s nutritional and health-promoting qualities. Additionally, the differential response between locations highlights the need for location-specific P management to maximize the benefits of sorghum cultivation in semi-arid regions.

## Supporting information

S1 Table Row data of all the genotypes and parameters, including replicates used in this study. (XLSX)

## References

[1] Serna-Saldivar S. and Espinosa-Ramírez J., Sorghum and Millets. 2019, Elsevier Amsterdam, The Netherlands:.

[2] Mohamed L.K., et al., Changes in Phytochemical Compounds and Antioxidant Activity of Two Irradiated Sorghum (Sorghum bicolor (L.) Monech) Cultivars during the Fermentation and Cooking of Traditional Sudanese Asida. Fermentation, 2022. 8(2): p. 60.

[3] Abdelhalim T.S., et al., Indigenous Sudanese sorghum‐based food: Secondary metabolites and antioxidant activities of traditional Sudanese nonalcoholic beverage Hulu‐mur from two sorghum landraces. Food Science & Nutrition, 2023. doi: 10.1002/fsn3.3275 37324862 PMC10261747

[4] DIRAR H.A., A microbiological study of Sudanese merissa brewing. Journal of Food Science, 1978. 43(6): p. 1683–1686.

[5] Xiong Y., et al., Cellular antioxidant activities of phenolic extracts from five sorghum grain genotypes. Food Bioscience, 2021. 41: p. 101068.

[6] Rao S., et al., Characterization of phenolic compounds and antioxidant activity in sorghum grains. Journal of Cereal Science, 2018. 84: p. 103–111.

[7] Jaćimović S., et al., Chemical composition, antioxidant potential, and nutritional evaluation of cultivated Sorghum Grains: A combined experimental, theoretical, and multivariate analysis. Antioxidants, 2023. 12(8): p. 1485. doi: 10.3390/antiox12081485 37627480 PMC10451854

[8] Girard A.L. and Awika J.M., Sorghum polyphenols and other bioactive components as functional and health promoting food ingredients. Journal of Cereal Science, 2018. 84: p. 112–124.

[9] Duodu K.G. and Awika J.M., Phytochemical-related health-promoting attributes of sorghum and millets, in Sorghum and millets. 2019, Elsevier. p. 225–258.

[10] Taylor J.R. and Kruger J., Sorghum and millets: Food and beverage nutritional attributes, in Sorghum and Millets. 2019, Elsevier. p. 171–224.

[11] Abdelhalim T., Jannoura R., and Joergensen R.G., Arbuscular mycorrhizal dependency and phosphorus responsiveness of released, landrace and wild Sudanese sorghum genotypes. Archives of Agronomy and Soil Science, 2020. 66(5): p. 706–716.

[12] Smith S.E., et al., Arsenic uptake and toxicity in plants: integrating mycorrhizal influences. Plant and Soil, 2010. 327: p. 1–21.

[13] Li H., et al., Biochar phosphorus fertilizer effects on soil phosphorus availability. Chemosphere, 2020. 244: p. 125471. doi: 10.1016/j.chemosphere.2019.125471 31809932

[14] Abbas Z., et al., Effect of phosphorus fertilizer and water stress on protein and phenolic contents in cotton (Gossypium hirsutum L.). Pakistan Journal of Agricultural Research, 2015. 28(4).

[15] Scagel C.F. and Lee J., Phenolic composition of basil plants is differentially altered by plant nutrient status and inoculation with mycorrhizal fungi. HortScience, 2012. 47(5): p. 660–671.

[16] Ma D., et al., Evaluation of yield, processing quality, and nutritional quality in different‐colored wheat grains under nitrogen and phosphorus fertilizer application. Crop Science, 2018. 58(1): p. 402–415.

[17] Yin X., et al., Phosphorus fertilization differentially influences fatty acids, protein, and oil in soybean. American Journal of Plant Sciences, 2016. 7(14): p. 1975–1992.

[18] Lux P.E., et al., Location and variety but not phosphate starter fertilization influence the profiles of fatty acids, carotenoids, and tocochromanols in kernels of modern corn (Zea mays L.) hybrids cultivated in Germany. Journal of agricultural and food chemistry, 2021. 69(9): p. 2845–2854. doi: 10.1021/acs.jafc.0c07571 33646789

[19] Talhaoui N., et al., Determination of phenolic compounds of ‘Sikitita’olive leaves by HPLC-DAD-TOF-MS. Comparison with its parents ‘Arbequina’and ‘Picual’olive leaves. LWT-Food Science and Technology, 2014. 58(1): p. 28–34.

[20] Waterhouse A., Polyphenolics. Handbook of Food Analytical Chemistry, 2005. 1: p. 461–470.

[21] Kim D.-O., Jeong S.W., and Lee C.Y., Antioxidant capacity of phenolic phytochemicals from various cultivars of plums. Food chemistry, 2003. 81(3): p. 321–326.

[22] Chang S.-T., et al., Antioxidant activity of extracts from Acacia confusa bark and heartwood. Journal of Agricultural and Food chemistry, 2001. 49(7): p. 3420–3424. doi: 10.1021/jf0100907 11453785

[23] Gülçin İ., et al., Determination of antioxidant activity of lichen Cetraria islandica (L) Ach. Journal of ethnopharmacology, 2002. 79(3): p. 325–329. doi: 10.1016/s0378-8741(01)00396-8 11849836

[24] Oraiza M., Studies on product of browning reaction prepared from glucosamine. Japanese J Nutr, 1986. 44: p. 307–15.

[25] Jayaprakasha G.K., Rao L.J., and Sakariah K.K., Antioxidant activities of flavidin in different in vitro model systems. Bioorganic & medicinal chemistry, 2004. 12(19): p. 5141–5146. doi: 10.1016/j.bmc.2004.07.028 15351397

[26] Re R., et al., Antioxidant activity applying an improved ABTS radical cation decolorization assay. Free radical biology and medicine, 1999. 26(9–10): p. 1231–1237. doi: 10.1016/s0891-5849(98)00315-3 10381194

[27] Price M.L. and Butler L.G., Rapid visual estimation and spectrophotometric determination of tannin content of sorghum grain. Journal of Agricultural and food chemistry, 1977. 25(6): p. 1268–1273.

[28] Jacques A., et al., Scientific note: bioactive compounds in small fruits cultivated in the southern region of Brazil. Brazilian Journal of Food Technology, 2009. 12(1/4): p. 123–127.

[29] Hetrick B., Wilson G., and Todd T., Mycorrhizal response in wheat cultivars: relationship to phosphorus. Canadian Journal of Botany, 1996. 74(1): p. 19–25.

[30] Vidal N.P., et al., The use of XLSTAT in conducting principal component analysis (PCA) when evaluating the relationships between sensory and quality attributes in grilled foods. MethodsX, 2020. 7: p. 100835. doi: 10.1016/j.mex.2020.100835 32195148 PMC7078354

[31] Tenenhaus M., et al., PLS methodology to study relationships between hedonic judgements and product characteristics. Food quality and preference, 2005. 16(4): p. 315–325.

[32] Pant A. and Pandey R., Bioactive phytomolecules and aging in Caenorhabditis elegans. Healthy Aging Res, 2015. 4(19): p. 1–15.

[33] Jan R., et al., Plant secondary metabolite biosynthesis and transcriptional regulation in response to biotic and abiotic stress conditions. Agronomy, 2021. 11(5): p. 968.

[34] Khan W., Prithiviraj B., and Smith D.L., Chitosan and chitin oligomers increase phenylalanine ammonia-lyase and tyrosine ammonia-lyase activities in soybean leaves. Journal of plant physiology, 2003. 160(8): p. 859–863. doi: 10.1078/0176-1617-00905 12964861

[35] Nell M., et al., Effect of phosphorus uptake on growth and secondary metabolites of garden sage (Salvia officinalis L.). Journal of the Science of Food and Agriculture, 2009. 89(6): p. 1090–1096.

[36] Yang C.Q., et al., Transcriptional regulation of plant secondary metabolism F. Journal of integrative plant biology, 2012. 54(10): p. 703–712. 22947222 10.1111/j.1744-7909.2012.01161.x

[37] Zhang J., et al., Transcriptome analysis reveals candidate genes related to phosphorus starvation tolerance in sorghum. BMC plant biology, 2019. 19: p. 1–18. 31296169 10.1186/s12870-019-1914-8 PMC6624980

[38] Gill S.S. and Tuteja N., Reactive oxygen species and antioxidant machinery in abiotic stress tolerance in crop plants. Plant physiology and biochemistry, 2010. 48(12): p. 909–930. doi: 10.1016/j.plaphy.2010.08.016 20870416

[39] Arora D., et al., Mechanisms of nitric oxide crosstalk with reactive oxygen species scavenging enzymes during abiotic stress tolerance in plants. Free Radical Research, 2016. 50(3): p. 291–303. doi: 10.3109/10715762.2015.1118473 26554526

[40] Chrysargyris A., Panayiotou C., and Tzortzakis N., Nitrogen and phosphorus levels affected plant growth, essential oil composition and antioxidant status of lavender plant (Lavandula angustifolia Mill.). Industrial Crops and Products, 2016. 83: p. 577–586.

[41] Wang Z., et al., Effects of phosphate-solubilizing bacteria and N2-fixing bacteria on nutrient uptake, plant growth, and bioactive compound accumulation in Cyclocarya paliurus (Batal.) Iljinskaja. Forests, 2019. 10(9): p. 772.

[42] Alam M.Z., et al., Effect of soil amendments on antioxidant activity and photosynthetic pigments in pea crops grown in arsenic contaminated soil. Heliyon, 2020. 6(11). doi: 10.1016/j.heliyon.2020.e05475 33241149 PMC7672278

[43] Talbi Zribi O., et al., Salinity and phosphorus availability differentially affect plant growth, leaf morphology, water relations, solutes accumulation and antioxidant capacity in Aeluropus littoralis. Plant Biosystems-An International Journal Dealing with all Aspects of Plant Biology, 2021. 155(4): p. 935–943.

[44] Chrysargyris A., et al., Effect of phosphorus application rate on Mentha spicata L. grown in deep flow technique (DFT). Food chemistry, 2019. 276: p. 84–92. doi: 10.1016/j.foodchem.2018.10.020 30409666

[45] Shen S., et al., Phenolic compositions and antioxidant activities differ significantly among sorghum grains with different applications. Molecules, 2018. 23(5): p. 1203. doi: 10.3390/molecules23051203 29772811 PMC6100422

[46] PANT B.D., et al., Identification of primary and secondary metabolites with phosphorus status‐dependent abundance in A rabidopsis, and of the transcription factor PHR 1 as a major regulator of metabolic changes during phosphorus limitation. Plant, cell & environment, 2015. 38(1): p. 172–187. 10.1111/pce.12378 24894834

[47] Hernández I. and Munné-Bosch S., Linking phosphorus availability with photo-oxidative stress in plants. Journal of experimental botany, 2015. 66(10): p. 2889–2900. doi: 10.1093/jxb/erv056 25740928

[48] Santos-Sánchez N.F., et al., Antioxidant compounds and their antioxidant mechanism. Antioxidants, 2019. 10: p. 1–29.

[49] Ślesak I., et al., The role of hydrogen peroxide in regulation of plant metabolism and cellular signalling in response to environmental stresses. Acta Biochimica Polonica, 2007. 54(1): p. 39–50. 17325747

[50] Noack S., McBeath T., and McLaughlin M., Potential for foliar phosphorus fertilisation of dryland cereal crops: a review. Crop and Pasture Science, 2010. 61(8): p. 659–669.

[51] Limmongkon A., et al., Antioxidant activity, total phenolic, and resveratrol content in five cultivars of peanut sprouts. Asian Pacific Journal of Tropical Biomedicine, 2017. 7(4): p. 332–338.

[52] Loganayaki N., Siddhuraju P., and Manian S., Antioxidant activity and free radical scavenging capacity of phenolic extracts from Helicteres isora L. and Ceiba pentandra L. Journal of food science and technology, 2013. 50: p. 687–695. doi: 10.1007/s13197-011-0389-x 24425970 PMC3671060

[53] Gülcin I., Antioxidant activity of food constituents: an overview. Archives of toxicology, 2012. 86: p. 345–391. doi: 10.1007/s00204-011-0774-2 22102161

